# Hospital and Institutional News

**Published:** 1918-11-16

**Authors:** 


### HOSPITAL AND INSTITUTIONAL NEWS.

### THE JAPANESE AT THE "DREADNOUGHT."

ANXIOUS to show the appreciation of his countrymen for the kind treatment given to Japanese seamen at the Seamen's Hospital, Greenwich, the Japanese Consul-General, Mr. K. Kamasaki, has induced Japanese merchants, shipowners, and shipbuilders to endow a bed at the institution. The fund quickly reached £1,000, and was gladly received by the hospital's chairman, Sir Perceval Nairne. The new bed will be happily called the "Cherry Blossom," and is a worthy companion to the "Chrysanthemum" bed already there.

### QUEEN ALEXANDRA AND SIR ARTHUR STANLEY.

QUEEN ALEXANDRA, in acknowledging the thanks of the Red Cross for her gift of £500 and her gracious messages in support of "Our Day," has written as follows to the Hon. Sir Arthur Stanley, Chairman of the Joint War Committee of the British Red Cross and the Order of St. John, 83 Pall Mall, London, S.W. 1. "How earnestly I echo your hope that this may be the last occasion on which it will be necessary to make a war appeal, but I agree with you that in the future the amount of work to be done will be immense. The Red Cross has, however, by its work, and I cannot help adding by its admirable administration, so completely gained the confidence of the whole English-speaking race that I think its efforts in the future will meet with the same generous and unbounded support that has always been extended to it in war time and in the past. This support has never been more needed, but it has never been greater or more splendid than during the past four years, when the calls upon the Red Cross resources have been almost unheard of in their extent, but which I am proud to think have always been met with a promptitude which has redoubled the value of its assistance."

### ITALIANS AND THE BRITISH RED-CROSS.

A TRIBUTE has been paid to the work carried on by the British Red Cross on the Italian front for more than three years by the presentation to the Hon. Sir Arthur Stanley, Chairman of the Joint War Committee of the British Red Cross and the Order of St. John, of the highest Order of the Croce al Merito of the Italian Red Cross. The insignia were brought in person to the British Red Cross Headquarters in Rome by the President of the Italian Red Cross, Conte Giuseppe Frascara. At the same time other grades of the Croce al Merito were bestowed upon Colonel Sir Courtauld Thomson, Chief Commissioner in Italy of the British Red Cross Society and the Order of St. John, and four other members of the British Red Cross Commission in Italy.

### A NEW USE FOR THE BOARD-ROOM.

MOST London hospitals have been affected by the influenza epidemic, both as regards staff and patients. Wherever one inquires it has been found that the nursing staff, who have run more risk of infection than anybody, have heavily suffered, and when a hospital nurse is warded the institution suffers doubly, for there is one more to nurse and one fewer to do the nursing. At the National Hospital for the Paralysed and Epileptic, Queen Square, and its branches, the ravages of the epidemic have been especially severe. When it was thought that the worst was over new cases occurred, and it was decided to limit the new outbreak by increased means of isolation. But all possible ordinary accommodation for isolation being already engaged, the management had to look around for a temporary ward elsewhere and decided, for the present, to make use of the large board-room of the hospital, which, despite unavoidable inconveniences, has proved most useful for the purpose. We wonder if there is any precedent for this novel use of the domains of the administrative department.

### AVENUES TO CULTURE.

WE are glad to note that the Educational Lectures for the Wounded at the Great Northern Central Hospital, in Holloway Road, have proved so successful that a second series is now being held. From "Diseases of Timber" to "Modern Poetry" and a lantern lecture on "Gold and Silver Broidery," by Miss M. Symonds, the first series wisely endeavoured to cover a varied field. The second series began on October 25 with Miss Mary Gaunt's account of her "Travels in Siberia," which were interrupted by the war in August 1914, and ended in her return to England by way of Russia. We hope that Mr. G. G. Panter, the hospital's secretary, or whoever arranges the lectures, will again attempt a varied course. Now that the end of the war appears to be near, the need for thought, for culture, for the balance of mind which avoids transports and hurried emotions, is indeed great. Most of us have been too busy "doing" to have time to "think," and the value of thought at a period of transition is to determine the end to which action should be devoted, and to lose itself in details of the "how" till it has settled the all-important "what" to be accomplished. These lectures are avenues to culture and balance of mind, and will be all the better if their subjects are not too familiar or too practical. Their aim should be to encourage judgment rather than to increase the information of their hearers.

### HONOUR FOR A MEDICAL SUPERINTENDENT.

THE French military authorities have presented Dr. Kerr, the medical superintendent of the Pilkington Orthopædic Hospital, St. Helens, with a medal in recognition of his services during 1915 and 1916 as surgeon and *médecin-chef* of the Alliance Hospital, Yvetot, in France. While Dr. Kerr was in charge of the institution an epidemic of diphtheria occurred in the district, to which he himself was a victim. The hospital is supported by Anglo-American funds.

### BEWARE OF PRUSSIAN LEGISLATION.

Now that the time is coming when the Insurance Act will again have to be revised, it is well to remember that it was made here on a German model. To see the system as it really worked there before the war, an impartial critic is desirable, and we therefore commend to institutional managers the views of Mr. Gerard, whose "Four Years in Germany" was mostly read on its appearance not for the information to be gleaned from an intelligent and impartial critic, but for the indictment which, in effect, it contained against the Prussian military party. Speaking of the Insurance system, Mr. Gerard said: "The working men in the cities . . . probably work longer and get less out of life than any working men in the world. The laws so much admired, and made ostensibly for their protection, such as insurance against unemployment, sickness, injury, old age, and so on, are in reality skilful measures which bind them to the soil as effectively as the serfs of the Middle Ages were bound to their masters' estates. I have had letters from working men who have worked in America begging me for a steerage fare to America, and saying that their insurance payments were so large that they could not save money out of their wages. . . . The working man naturally hesitates to emigrate and so lose all the premiums he has paid to the State." That is the case against *compulsory* State Insurance in a nutshell, and though we are probably committed to it now, when the time for revision comes it may serve to remind us to beware of extending the sphere of State compulsion. We must remember that the failure of this insolvent Insurance Act has led to the demand for a Ministry of Health. This Ministry, unless watched, may prove as inimical to the voluntary hospitals as the Insurance Act has been to the free choice of doctors by the poor.

### THE RISKS OF "RECONSTRUCTION."

The evils of compulsory State institutions, so impartially exposed by Mr. Gerard in his much cited but little read book, "My Four Years in Germany," deserve careful study by all believers in the voluntary system of England. He says in effect, that what we should call the Poor-Law taint of pauperism envelops all the State institutions to which the German is subjected. A noteworthy sentence in the chapter called "The System," runs: "The Germans are taken care of and educated very much in the same way that the authorities here look after the inmates of a poorhouse or penitentiary." What a picture this calls up! How it warns us, too, as believers in the voluntary system, to scrutinise carefully the ubiquitous proposals of Dr. Addison, and to search thoroughly the nature and effect of all the suggestions which the electors will be asked to applaud under what has grown to be the specious name of "Reconstruction"! It remains to be seen how far the returned soldiers and the new women voters will fight for the voluntary system of the hospitals which we have, and which gave us our first Army, or how far they may be willing to accept the system of German enslavement which lies behind so many proposals for "social reform." The cry should be: Remember the Insurance Act, which was made in Germany!

### TWO NEW COTTAGE HOSPITALS.

Under the leadership of Colonel Campbell, of Stanfield, a committee has been formed to provide a cottage hospital in Tarbert, near Oban. The late Captain Reid, of Tarbert, left a legacy of £2,000 for the purpose, and many others have interested themselves in the plan, which will probably be the memorial to the men of the district who have fallen in the war. Miss MacMichael is the honorary secretary of the committee. A similar movement has made a start at Leyton and Leytonstone, where a sub-committee, entrusted with the details, is thinking out ways and means, establishment, and maintenance.

### A NEW SANATORIUM FOR THE MIDLANDS.

The Kinver Edge Hotel has been taken on lease by the Staffordshire, Wolverhampton, and Dudley Joint Committee for Tuberculosis, to be used as a sanatorium. The "Limes," Himley, has also been acquired by the same authority for a similar purpose. The sites of both have been commended, and when the necessary alterations shall have been made, are considered to be well adapted for sanatoria.

### THE "BALKAN BED."

Formed to supply surgical appliances, in the widest sense, the organisation of inventors and makers known as the Surgical Requisites Association is a not widely known but very active body with branches all over the United Kingdom. Its work is carried on by women, and supported by voluntary funds: it is, in fact, the orthopædic branch of Queen Mary's Needlework Guild. From its pleasant premises at 17 Mulberry Walk, Chelsea, has issued a model guide to its own doings, in the course of which readers are invited to visit the Association's numerous workshops, and to see the ingenuities of the surgical aid studios. Among these is the S.R.A. Balkan Bed, which, as many have by now learnt, can be raised as high as six feet from the floor, so that the surgeon or nurse, by removing one of its sections, may expose a desired region. At will, too, the bed can be raised at either end to decrease blood pressure or assist drainage. The cost is £25. Many of the workers are voluntary. That in itself is a proud claim which deserves the support of intelligent people.

### THE AMERICAN EMIGRANTS FROM WALES.

Major David Davies, M.P., has offered, free of rent, charges, and conditions on its use, to the United States Army Medical Authorities the fine Montgomeryshire manor-house of "Greg-y-Nos," suggesting that it would be a suitable convalescent home for those American soldiers who are Welsh emigrants, of whom there are stated to be many thousands. "Greg-y-Nos" is noted for its 365 windows.

### PENSIONS AND TUBERCULOSIS: A NEW IDEA.

Even that venerable institution the law has always needed what is technically known as equity to modify its decisions, and in similar fashion it is easily to be seen that no matter how carefully and skilfully war disabilities may be medically assessed, some obvious misfits in the way of pensions are bound to result. Various schemes have been proposed to adjust hardship on the one hand with windfalls on the other. An officer invalided for (presumably) tuberculosis states, in a letter to a lay contemporary, what he would have done for discharged consumptives. Where the earning capacity brings an amount more than equal to the pension, such excess should be taken—by a committee nominated by the Pensions Ministry—and devoted to making up the earnings in those cases where they fall below the amount of the pension. If such receipts are insufficient for the latter purpose, the Treasury should make good the deficit. The officer also urges that invalids refusing to take work suited to their capacity and physical condition should be compelled to do such work under pain of forfeit of or reduction in pension. It is a clear and definite proposal, but the test of all such schemes cannot be other than practical; and to compel a man to accept work which *someone else* deems to be "suited to his capacity" is rather too Prussian a solution for many of us.

### LEASED PREMISES FOR TUBERCULOUS CASES.

Local authorities are asked by the Local Government Board to find more accommodation for tuberculous patients. It is suggested that small-pox hospitals might be made available by providing elsewhere for sporadic cases of small-pox. The Local Government Board intimates that, if necessary, it will sanction the leasing of any suitable premises. Some of the extra accommodation urgently required can be provided only by the extension of existing institutions.

### THE NEW PUBLICITY.

The publicity department of the voluntary hospital has a double duty, one half of which, we venture to say, is still unperformed. Its first duty is to use with skill and discretion every means to make its work known. This has become generally understood, so that an up-to-date hospital "appeals" to the public more by the announcement of its doings than by the description of its needs. The consequence has been that every column of the newspapers is now open to the alert secretary: for the appeal lies to-day as much in the interest aroused as in the sympathy excited. Thus the newspaper world is now virtually at the call of the voluntary hospital. The art of publicity has been mastered, and hospital men have become recognised and valued contributors to the press. This has laid upon them an obligation corresponding to the new advantage gained. It is an obligation which, we fear, is still neglected, an obligation to the body of men through the agency of which the power of journalism is at their disposal—namely, to the newsvendors. These men, who perform a necessary and an underpaid service, are the Cinderellas of journalism. Their one prop and stay is the Newsvendors' Institution, at 15 Farringdon Street. It looks after those of its members who are in distress, and they are often numerous. It gives pensions, on as generous a scale as it is able, in return for small payments. Since 1913 the contributed income has fallen to one-third of the figure then reached, and that was a modest one. The single thin sheet, folded in half, on which the latest report is issued, is a startling proof of the need for economy. Hospital managers, who owe an enormous debt to their humbler friends the newsvendors, should make it a point of honour to become annual subscribers to the Newsvendors' Institution. They owe the men whom it serves a debt of honour, a debt they should be eager to repay.

### THE SPEECH CLINIC AT ST. THOMAS'S.

The Central School of Speech Training has had to be moved from the crush-rooms at the Albert Hall, now commandeered, to another part of the building and to the Imperial Institute. The Speech Clinic at St. Thomas's Hospital is understood, however, to be unaffected and will continue until a separate building in an accessible part of London can eventually house the Central School permanently. It may even then persist. Meantime, however, far too little is generally known of the aims and work of the Speech Clinic at St. Thomas's Hospital, and we wish that the ever-courteous secretary would educate the public in what is being done.

### A COTTAGE HOSPITAL FOR SHEERNESS.

In consequence of the departure of the vicar of Holy Trinity, Sheerness, the Rev. A. D. Vernon, on military service, the scheme for a cottage hospital for the port and island is being realised. The vicar kindly offered Alexandra House, the vicarage, for use as a cottage hospital at the nominal rent of 10s. per week. So that the original idea, which Mrs. Hyde-Smith, the wife of the Admiral-Superintendent started, for the time being at all events, holds the field. Under the influence of the dockyard Labour representatives the local authorities were considering the matter, but voluntary effort once again has moved faster than official machinery. Luckily too the War Emergency Fund committee, which Mrs. Hyde-Smith had approached, has received several donations reserved for a local cottage hospital; and, as was fitting, Mrs. Hyde-Smith has been elected president of the hospital committee, to which Mr. W. J. Wood is acting as the honorary secretary. The Carnival Committee, arranged by the Dockyard Workers, has handed £330 on behalf of the cottage hospital to the new president. So the latest cottage hospital is making an encouraging start.

### PAY WARDS AT LEICESTER ROYAL INFIRMARY.

The suggestion of the Rev. Arnold Manvell, Vicar of St. Barnabas, Leicester, that advantage should be taken of the proposal to rebuild the old part of the Royal Infirmary by including pay wards therein was worth making. As he said lately in the *Leicester Mercury*, "Now is the time to discuss the idea before the plans are finally settled. We cannot doubt what the ultimate decision will be. To complete its development the Royal Infirmary should certainly possess sufficient wards for paying patients. Can they, then, not be included in the proposed rebuilding scheme?

### THE CROSS ON THE TRUNK.

The latest thing to be sold for the benefit of the Red Cross is a forest of trees, each provided by a different landed owner. Trees for felling are usually marked with a white cross. If still more must be felled, alas, let it be the Red Cross to claim them. An oak from Windsor Park is the first.

### THE RISK OF CONTINUOUS PERFORMANCES

In view of the prevalence of influenza, modern picture palaces cannot be classified as health resorts. In spite of some endeavours to prohibit continuous performances at cinemas, at some of them the show proceeds all the afternoon, evening and night.

### PLUMS FROM AN AMERICAN PUDDING.

Many excellent things are packed away in the *American Red Cross Bulletin*, which is admirably edited. We take the following from a recent number: The Commission in Belgium, in a report that the American Red Cross and Maternity Hospital and Children's Dispensary at Leysele, near the Belgian front, was lately bombed by German aeroplanes, restrained by remarks that only "eight bombs were dropped. Fortunately a few women only were wounded." An interesting illustration is given of the new pneumonia ward at Mossley Hill Hospital, Liverpool, which was finished in time to be fully useful during the epidemic. News that the American Red Cross laundry at Southampton was nearly stopped for lack of coal happened to reach a group of American sailors. They learned on asking that it was six miles away, and that there was no lorry in which to fetch it. The men procured a Navy truck, which within three hours was unloading twenty tons at the laundry door.

### AN AMERICAN SENTIMENT.

Americans have little ways as well as infectious energy. One of the former was noticed by Mrs. Arthur Robinson, of Baltimore, in the course of her work as Director of Women Visitors at Dartford Hospital. She informs the *Bulletin* that the men, when asked about their next-of-kin, usually mention their mothers. One, when pressed, said that he mentioned his mother, not because she was a widow, but because, in spite of his father's vigorous health, his mother was "of course" his "nearest relation." What, we wonder, do these believers in matriarchy make of the famous reply to the same question, the reply "Who is my mother?" or is this phrase now revised, on modern lines, in the American Version?

### WAR BONUS FOR MARRIED MEN IN THE ARMY.

In consequence of a request made by the staff of the Metropolitan Asylums Board that married men serving with the Forces should receive consideration for the benefit of their families, an interesting recommendation has been made. The General Purposes Committee of the Board lately proposed that in calculating the difference between the civil pay of employees absent with the Forces and the Army or Navy pay, no account should be taken of the separation allowances. Though the Board accepted this recommendation, there was a legal difficulty in carrying it through. Therefore the Local Government Board preferred that the example of many local authorities should be followed by giving to married men or those with dependants absent on military duty the benefit of the war bonus as part of their civil remuneration. In other words the phrase "full pay of any rank" are now to be held to include, in the case of married men or those with dependants, the appropriate war bonus of the particular grade.

### THE NEW "WELFARE" POWERS.

Local authorities are already preparing to use the new powers conferred by the Maternity and Child Welfare Act. Willesden Urban Council has asked the medical officer of health to prepare a scheme for a convalescent home for children with fifty beds. Hammersmith Borough Council has asked the medical officer to prepare a scheme for a lying-in hospital, to include provision for home helps and so forth.

### THIS WEEK'S DRUG MARKET.

As might be expected, the drug market is extremely quiet at the moment. This is not only because dealers and buyers are too busy celebrating peace to think of business, but because everyone is waiting to get some indication of how far prices are likely to recede. At present there is no indication as there is practically no business. But when we consider that buying on behalf of the Medical Services of the Armies will be much reduced, that labour will soon be more plentiful, that ships will take a much shorter time to cross the seas and will be sure of reaching their destination, it seems impossible to believe that prices generally can be maintained. Therefore, the wise buyer will keep off the market for everything which he does not immediately need. The restrictions on glycerin are about to be removed. The Government has fixed prices for quinine at figures about half those ruling a week ago.

